# [Retracted] SET and MYND domain‑containing protein 3 is overexpressed in human glioma and contributes to tumorigenicity

**DOI:** 10.3892/or.2024.8825

**Published:** 2024-10-15

**Authors:** Bin Dai, Weiqing Wan, Peng Zhang, Yisong Zhang, Changcun Pan, Guolu Meng, Xinru Xiao, Zhen Wu, Wang Jia, Junting Zhang, Liwei Zhang

Oncol Rep 34: 2722–2730, 2015; DOI: 10.3892/or.2015.4239

Following the publication of this paper, it was drawn to the Editor's attention by a concerned reader that certain of the colony formation assay data featured in Fig. 4A and C on p. 2726, tumor images in Fig. 6A on p. 2727, and western blotting data shown in Fig. 7A on p. 2728 were strikingly similar to data that had appeared in other articles written by different authors at different research institutes, which had already been published before this article was received at *Oncology Reports*. Owing to the fact that the abovementioned data had already been published prior to the receipt of this article at *Oncology Reports*, the Editor has decided that this paper should be retracted from the Journal. The authors were asked for an explanation to account for these concerns, but the Editorial Office did not receive a reply. The Editor apologizes to the readership for any inconvenience caused.

